# The notes from nature tool for unlocking biodiversity records from museum records through citizen science

**DOI:** 10.3897/zookeys.209.3472

**Published:** 2012-07-20

**Authors:** Andrew Hill, Robert Guralnick, Arfon Smith, Andrew Sallans, Rosemary Gillespie, Michael Denslow, Joyce Gross, Zack Murrell, Tim Conyers, Peter Oboyski, Joan Ball, Andrea Thomer, Robert Prys-Jones, Javier de la Torre, Patrick Kociolek, Lucy Fortson

**Affiliations:** 1 Vizzuality, New York, New York, USA; 2 University of Colorado, Boulder, Colorado, USA; 3 Adler Planetarium, Chicago, Illinois, USA; 4 University of Virginia, Charlottesville, VA, USA; 5 University of California Berkeley, Berkeley, California, USA; 6 Appalachian State University, Boone, North Carolina, USA; 7 Department of Zoology, Natural History Museum, Cromwell Road, London SW7 5BD, UK; 8 University of Illinois, Urbana-Champaign, Champaign, Illinois, USA; 9 Bird Group, Natural History Museum at Tring, Akeman Street, Tring, Herts HP23 6AP, UK

**Keywords:** Natural History Museums, Biodiversity, Open Source, Museum Collections, Citizen Science, Digitization, Transcription

## Abstract

Legacy data from natural history collections contain invaluable and irreplaceable information about biodiversity in the recent past, providing a baseline for detecting change and forecasting the future of biodiversity on a human-dominated planet. However, these data are often not available in formats that facilitate use and synthesis. New approaches are needed to enhance the rates of digitization and data quality improvement. Notes from Nature provides one such novel approach by asking citizen scientists to help with transcription tasks. The initial web-based prototype of Notes from Nature is soon widely available and was developed collaboratively by biodiversity scientists, natural history collections staff, and experts in citizen science project development, programming and visualization. This project brings together digital images representing different types of biodiversity records including ledgers , herbarium sheets and pinned insects from multiple projects and natural history collections. Experts in developing web-based citizen science applications then designed and built a platform for transcribing textual data and metadata from these images. The end product is a fully open source web transcription tool built using the latest web technologies. The platform keeps volunteers engaged by initially explaining the scientific importance of the work via a short orientation, and then providing transcription “missions” of well defined scope, along with dynamic feedback, interactivity and rewards. Transcribed records, along with record-level and process metadata, are provided back to the institutions.  While the tool is being developed with new users in mind, it can serve a broad range of needs from novice to trained museum specialist. Notes from Nature has the potential to speed the rate of biodiversity data being made available to a broad community of users.

## Introduction

Natural history collections represent irreplaceable legacy information about our biosphere. In an era dominated by planetary-scale anthropogenic change (Walther et al. 2002, Parmesan and Yohe 2003) and unprecedented biodiversity loss (Jenkins 2003, Loreau et al. 2006, Wake and Vredenburg 2008), both historical and recent biocollections and their associated data represent valuable benchmarks for analyzing the biological impacts of environmental change and determining its causal factors (Moritz et al. 2008, Rainbow 2009, Pyke and Ehrlich 2010, Erb et al. 2011). The knowledge derived from specimens has been a critical component in studies of invasive species (Giovanelli et al. 2008, Rödder and Lötters 2009); biological conservation (Pawar et al. 2007); land management (Ochoa-Ochoa et al. 2009); pollination (Biesmeijer et al. 2006); species distributional (Lyons and Willig 2002, Peterson 2003, Moritz et al. 2008, Peterson and Martínez-Meyer 2009) and phenological (Nufio et al. 2010) responses to climatic change; spread of pathogenic organisms (Moffett et al. 2009, Soto-Azat et al. 2010); species discovery (Bebber et al. 2010); and forecasting future changes (Graham et al. 2004).

It is estimated that the number of specimens in natural history collections could range anywhere from 1 billion for just arthropods (Nishida 2003) to 2 billion records for all collections (Ariño 2010). Whatever the final number, the current representation of digitized records is much less. The Global Biodiversity Information Facility (GBIF) maintains the largest single portal to digital species occurrence records -- currently provisions about 400 million records, many of which are from citizen observation networks and not natural history collections. Further, the taxonomic representation in GBIF is skewed to those taxonomic communities and regions of the world where support for digitization has been strongest. While the current digital available representation of vertebrates in Western Europe and North America may be quite good, for groups such as insects in regions such as the tropics, our data remain particularly limited (Guralnick and Hill 2009). Biocollections contain abundant historical records (Boakes et al. 2010) that help fill the gaps from early time-periods, often pre-dating massive human-caused changes to landscapes. Furthermore, these collections often contain important biological records that can help further the study of biodiversity today (Pyke and Ehrlich 2010).

Despite the well-documented value of biocollections for science and society, the ability of researchers and policy makers to utilize this resource is hampered because many specimen data remain sequestered within institutions in non-digital formats. Digitization, transcription, description, and mobilization of specimen data (including label data, images, field notes, illustrations, and gene sequences) improves data discovery, interoperability, and enhancement (Edwards et al. 2000, Canhos et al. 2004, Soberón and Peterson 2004, Guralnick and Hill 2009), but these activities are not automatic, and present technical and organizational challenges (Pennisi 2005, Berendsohn and Seltmann 2010).  Many institutions lack the financial, technological, or staffing resources needed to complete the many tasks required to deliver well-described digital data to data consumers (Vollmar et al. 2010). Even those institutions fortunate enough to have the needed resources and capacity may still want to utilize new methods that engage the public, serve educational missions, and potentially deliver more error free data while also scaling down total digitization costs.

Specimen digitization (i.e. digitally capturing each component of the specimen label and at times the specimen) is a multi-step process, and one of the most expensive and time-consuming of those steps is transcribing the labels into textual formats essential for further description and querying. This is particularly challenging when labels are hand-written, rendering other techniques such as optical character recognition (OCR) mostly useless. While OCR can prove valuable with printed or typed labels, and will undoubtedly play an important role in the future, the technology is still prone to errors that need to be corrected and validated. There is, however, a potentially transformational solution to this problem: working with citizen science volunteers across the world to help with transcription tasks.

Citizen science, where volunteer researchers are asked to help create or process scientific data, is becoming popular on the web (Zooniverse, https://www.zooniverse.org/; Folding@home, http://folding.stanford.edu/ ) and in web-enabled field collection (eBird, http://ebird.org/; iNaturalist, http://inaturalist.org/ ). Biological specimen transcription is a task well suited for citizen science, and a small number of projects have already been developed. Herbaria@home (http://herbariaunited.org/atHome/ ) for example, provides a portal to the herbarium sheets from primarily the United Kingdom and Irish herbaria. The work done by Herbaria@home has helped unlock over 100,000 specimens, making them digitally available for further science research. A more recently launched project, Atlas of Living Australia (ALA) Biodiversity Volunteer Portal (http://volunteer.ala.org.au/ ), has a broader scope, digitizing records and field notes from Australia’s biodiversity collection. The ALA site builds missions and encourages users to earn badges for their efforts. The Volunteer Portal has brought in around 200 volunteers who have completed nearly 20,000 transcription tasks.

**Figure 1.**
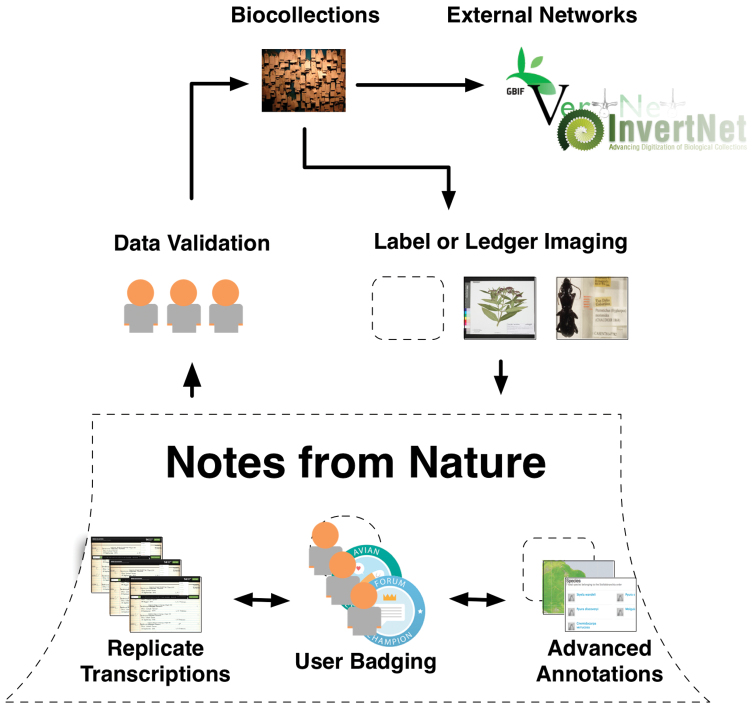
Organization of the Notes from Nature platform.

Here we describe for the first time a prototype citizen science application for transcribing cross-institutional, taxonomically diverse, natural history ledgers and labels called *Notes from Nature* (http://www.notesfromnature.org/; Figure 1). In describing this tool and how it was designed, we hope to also provide insights into data management and quality assurance methods, volunteer engagement practices, and education and reward mechanisms in online citizen science project development. We frame our development process using knowledge and tools gained from other Zooniverse projects, which has pioneered web-based citizen science in other disciplines, while discussing unique aspects of working with natural history specimen based image sources. In particular, we discuss topics important to the development and management of citizen science applications, such as methods to provide user feedback, communication and rewards to volunteers, and testing accuracy compared to more traditional transcription practices.

## Methods and results

### Data resources for initial phase of notes from nature

Notes from Nature is currently in a prototype phase and was developed in a collaboration between institutions and consortium including: Natural History Museum London bird collection (NHMUK; http://www.nhm.ac.uk/research-curation/departments/zoology/bird-group/index.html ), the Southeast Regional Network of Expertise and Collections (SERNEC; http://www.sernec.org/ ) organization, Calbug (http://calbug.berkeley.edu/ ), and the University of Colorado Museum (http://cumuseum.colorado.edu/Research/Zoology/ ). The NHMUK contributes an iconic group of organisms with a long history of enthusiasts and volunteer communities ‒ birds. SERNEC is a collaboration of Southeastern United States herbaria to bring collections “online” in part through digitization efforts of herbarium sheets. Calbug is a collaboration involving multiple entomological collections in California and coordinated by the University of California Berkeley’s Essig Museum of Entomology (EMEC); one goal is to provide a model for the digitization of diverse and digitally underrepresented arthropod specimens. The University of Colorado Museum of Natural History (UCMNH) is providing a unique validation dataset discussed in more detail below.

**Figure 2.**
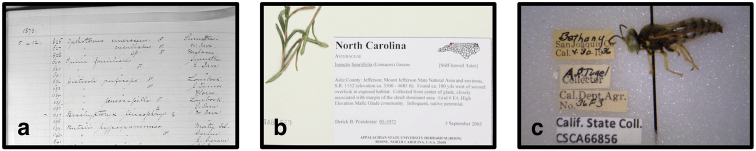
Example biocollections source images showing (**a**) The Natural History Museum, London bird specimen ledger; (**b**) The Southeast Regional Network of Expertise and Collections herbarium sheet label; **(c**) Calbug specimen and label image.

The input data and images from these three groups fall into three different categories. The NHMUK data consist of images of hand-written ledger pages that contain each component of a record organized in rows and columns (Figure 2a). SERNEC provides images of plant specimens with associated labels: in this case, specimens are flat, and are therefore particularly amenable to photographing, and suffer minimal image loss or distortion in the third dimension (Figure 2b). The Calbug digitization processes are particularly challenging because individual specimens are mounted, along with labels, on pins (Figure 2c).  Each specimen is carefully removed and photographed alongside each associated label. The three projects have independent, and for SERNEC and Calbug, ongoing imaging initiatives that are driving content for Notes from Nature.

We have collected an additional 100 images, representing ledger pages of bird specimens containing over 1000 records from UCMNH, to be used as reference standards. The full set of these records has already been databased once, creating an objective standard of quality for comparison. These images were then re-transcribed by trained museum staff in Fall of 2011 using current best practices in order to calculate rate and current cost. The transcription of these records will then also be duplicated by Notes from Nature volunteers. Local “staff” and citizen science retranscriptions will then be compared to the original datasets in order to generate statistics regarding accuracy, speed, and required training of the volunteer community to create data on the Notes from Nature platform. We will make such statistics publicly available on the Notes from Nature blog. We note that this initial comparison, although useful, may not generalize to other types of material (e.g. herbarium sheets, specimen labels). However, such initial statistics are of high value given only anecdotal information by which to judge cost efficiency and quality. Further such tests can only help provide assessment of the cost and quality effectiveness of the citizen science approach.

### Notes from nature platform design overview

Notes from Nature is being developed with personnel and programming support from The Citizen Science Alliance (CSA; http://www.citizensciencealliance.org/ ), which develops and maintains a roster of projects called the Zooniverse (http://www.zooniverse.org/ ), and Vizzuality (http://www.vizzuality.com/ ), a CSA parter that specializes in biodiversity visualization. A core team of CSA developers, designers and educators is funded by a grant from the Alfred P. Sloan Foundation that promotes the development of new citizen science projects at the Zooniverse. Zooniverse projects are growing in diversity but each project builds upon a set of technologies that aid common features across projects such as transcription data collection and user communication (https://github.com/zooniverse ).

**Figure 3.**
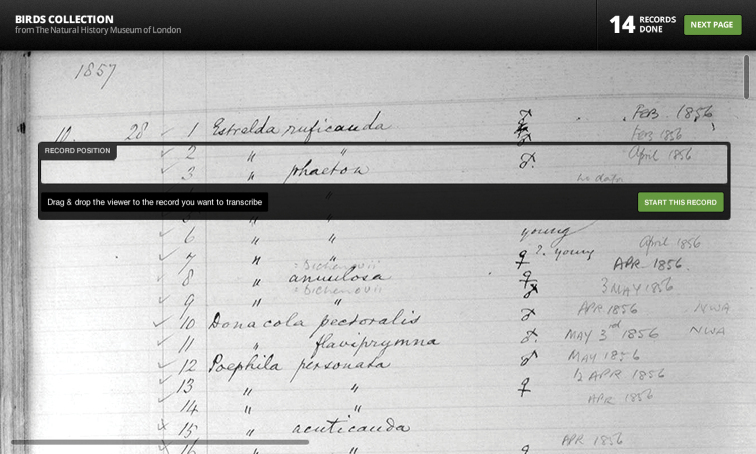
The Notes from Nature transcription tool for NHMUK museum ledgers. The tool gives users basic methods to navigate through a page of collections records while transcribing each major component of the record, viewing help dialogs, or skipping difficult to transcribe record entries. For help dialogs, we provide more than one example for each record element. The record outline is a movable window and, during transcription, the image and the tool location on that image is also captured as metadata, so that data managers can return quickly return to the source material for any record.

The front end of the platform is built on a stack of the latest web-technologies using JavaScript and HTML5. The transcription tool, for example, uses a mix of HTML5 Canvas and JavaScript to give the user a simple mechanism for capturing each record’s location and content. The system is designed to have different user-interfaces tailored to the image layout and information displayed. For example, the transcription tool layout for row-and-column based ledger page images (Figure 3) will differ from the layout for mounted plant specimen and label images. The tool is open-source and code is available online at https://github.com/Vizzuality/BioTrans .

The design of Notes from Nature takes it cues from other successful Zooniverse projects. Any person with Internet access can create a Zooniverse account and join the project (or any other project in the Zooniverse). Prior to performing any transcription, a new user is led through a short series of tutorials. These demonstrate the process of accurate transcription, but more importantly explain how and why the data are important to scientists.  In previous Zooniverse projects, orientation tutorials have proven especially valuable for imparting the urgency and value of the work which in turn provides initial motivation for involvement (Raddick et al. 2010).

Notes from Nature organizes the raw data – digital images – in three different ways: by projects, by collections, and by missions. “Projects” are large, unified, datasets provided by partner museums or consortiums or museums. SERNEC and Calbug are two distinct examples of projects. “Collections” are the organizing subunits within projects. For example, Calbug is a collaboration across eight different institutions, and each institution that has records in Notes from Nature will be referred to as a “collection”. The three projects are shown on different pages of the Notes from Nature site so that volunteer transcribers can learn about the projects and collections that interest them them most. While the real world organization of projects and partners can be complex, the simplification is intended to help users find relevant information about the specimens they are transcribing. Finally, the Notes from Nature team is developing “missions” that thread narratives across or within projects and collections. Missions are meant to engage the users, especially those with special interests in a particular organism or group of organism (e.g. beetles) or regions (e.g. west African tropics). Each missions has a clear end-point, where every record in the mission is transcribed or determined to be too challenging for transcription and the mission is considered complete.

During the transcription process on Notes from Nature, the user examines and transcribes records or ledger pages one at a time. The work a user performs is recorded, and elements of that work will be displayed as part of their personal profile page; a user’s personal data may include what collections they have worked, how many missions in which they have taken part, or on what missions they are currently working. As discussed below in more detail, transcribers are also rewarded for completing certain kinds of tasks, acquiring badges for different kinds of activities such as completing a certain number of records in a particular taxonomic group or geographic area, finding new and unusual records such as previously unrepresented species of organisms.

### Transcription and storage of results using notes from nature

The transcription tool is the workhorse of Notes from Nature, capturing both text inputs from the user along with its own position and the page on which it is being used. Volunteers move the tool to overlap a single specimen record among the many on a ledger sheet, and then transcribe and categorize the components of each record, such as collector, geographic, temporal, and taxonomic fields. In all cases, a record of the image or page of the scanned material, the record’s identification in a collection or project, and the location of the transcription on the digital image are stored in a MongoDB back end hosted by the Citizen Science Alliance.

**Figure 4.**
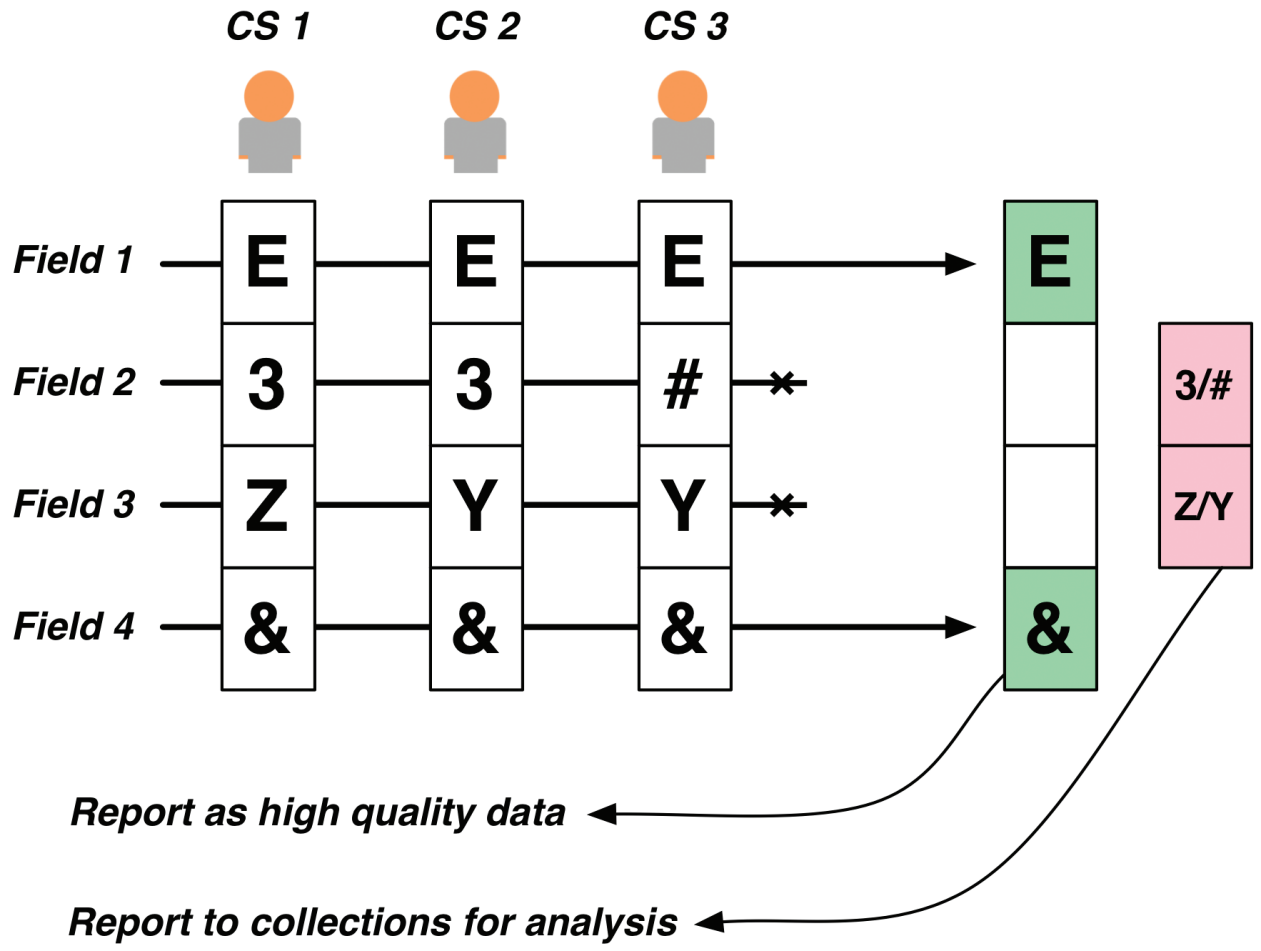
The simplified transcription replication and validation step. Following three independent transcriptions of a record, data is reconciled and returned to the original data provider. Records sent back to the provider can be fully complete, partially complete, of fully incomplete. Fully complete records are those where all three citizen scientist volunteers (CS) agree on every field of the record. Partial records include only those fields where CS agree. Fully incomplete records indicate that volunteers were largely unable to transcribe the record consistently. Data collected that does not become part of the final record is still made available for further review by the data provider.

The accuracy of transcriptions generated in Notes from Nature is evaluated by collecting at least three replicate transcriptions for every record (Figure 4). The level of convergence by volunteers is used to evaluate confidence in the output (Lintott et al. 2008). The accuracy for each field within a record (such as date of collection or species name) can be measured independently, allowing trained staff to then revisit problematic records and work to resolve discrepancies outside of the Notes from Nature platform.

The full record collected at transcription, including all multiple replications, are returned to the original data providers as both “raw” outputs and summaries that can provide quick views of progress (number of records transcribed on a day, total hours spent, etc). Notes from Nature will assure that the core fields, and other parts of records that are valuable to collect but might be idiosyncratic to a collection, meet community standards (Wieczorek et al. 2012). We will ask all users to transcribe records verbatim. The task of the citizen scientist is not to correct the original data, but instead to make it digitally available. In later versions of Notes from Nature, we plan to include interfaces for advanced users to suggest corrections to the original record. Part of this future work will be cleaning records to conform to the controlled vocabularies in standards such as Darwin Core.

For the Notes from Nature initial prototype, the goal is to assure that the essential fields of each partner institution are captured verbatim, with metadata about collection and replication. Core members of the Zooniverse and Vizzuality teams will be working with the project leads to ensure the data is captured effectively and returned to the home institutions in formats most useful for further integration back into databases. As per collaboration agreements, all data collected from this project will be made freely available online in usable formats (e.g. Darwin Core records) by the collaborating projects (NHMUK, SERNEC, Calbug) or their member institutions.

### Volunteer engagement and incentives

The methods for engaging volunteers in the Notes from Nature project can be categorized in three ways: communication, transcription feedback and narratives, and incentives.

Communication: Notes from Nature, like most projects on Zooniverse, encourages users to interact with both scientists and other volunteers in a purpose-built discussion platform (https://github.com/Zooniverse/Talk ) and via live-virtual discussion. The live discussion interfaces serve as an excellent medium for comments and questions and also become a focal point of communication to and from the researchers that are interested in seeing this data inform future science and conservation. Like other CSA projects, Notes from Nature will have a blog for communicating and archiving major news, discoveries, and milestones to the community. The blog will also become a tool for outreach, seeking new volunteers from existing clubs and communities.

Transcription feedback and narratives: Notes from Nature will provide immediate information about how a user’s actions are expanding the library of information for scientific research. Records transcribed can be shown as part of a “collective map” illustrating how new records streaming in from all Notes from Nature volunteers are closing gaps in our knowledge. Similarly, users will be given data-driven narratives such as collector histories, where we will create maps showing where collectors have travelled, telling small stories about the scientific work and contribution of the people who helped create the biological collections. Users will also get feedback about the taxa they are transcribing utilizing taxon resolvers and displaying content such as images or narratives from EOL and Wikipedia in the Notes from Nature interface.

Incentives: Users will receive badges that are marks of accomplishment that can be kept on the Notes from Nature site and shared with others broadly via other social media sites. Distributing digital badges to represent new skills or achievements and thus promote learning and further engagement is a trend emerging in education fields (Goligoski 2012); however, rigorous studies demonstrating whether or not badges enhance citizen science motivation and learning have yet to be performed. Examples of badges in Notes from Nature may include “World Explorer” for those who complete transcriptions in a large number of countries, or “Bird Expert” for those who transcribe the top number of bird records.

## Conclusion

The development of web-based citizen science endeavors stems from a long tradition of utilizing volunteers with a strong interest in the scientific subject matter (Cohn 2008). Such volunteer work has typically taken place locally at museums or other institutions, but the rise of the World Wide Web has provided a new, global platform for unpaid citizen efforts (Cravens 2000). Citizen science projects have taken many forms, the most well known among the biology community being outdoors-based reporting of species geographic distribution (e.g. iNaturalist, eBird; Sullivan et al. 2009) and phenology (e.g. Project Budburst; Meymaris et al. 2008). These projects are facilitated by the Internet, but have their roots in citizen volunteer efforts that, in cases like the Christmas Backyard Bird Count, stretch back more than a century.

A new category of citizen science leverages the Internet to disperse, transform, and reassemble information at unprecedented rates. These citizen science projects focus less on the creation of new scientific records, and more on the interpretation or enhancement of existing data sources and grow from a legacy of online volunteer transcription and proofreading started over a decade ago (See Distributed Proofreaders, http://www.pgdp.net/ ). Transcription of natural history collections records is a particularly strong fit for this new form of web-enabled citizen science, given the scope of the challenge, the scientific need for these data, and the inherently interesting subject matter. Other projects attempting similar outcomes are underway, including the Atlas of Living Australia Biodiversity Volunteer Portal and Herbaria@home, but each of these vary from Notes from Nature in scope and the tools deployed. However, with existing projects in place and future projects being considered, a key question is whether the approach will capture the imagination of enough people to remain a reasonable, cost-effective and long-term solution to the challenge of transcribing as many as a billion objects.

Citizen Science on the web is in its infancy, and our knowledge about what works and why is still developing. The methods and product we are developing for Notes from Nature are helping to expand and build upon that knowledge. In particular, working within the Zooniverse offers experience with a legacy of technological tools, such as live-chat and reusable back-ends, a consistency across citizen science projects, and a strong focus on understanding and replicating successes while avoiding pitfalls. As importantly, the Zooniverse has generated a critical mass of volunteers and has established itself as a key member in the community creating citizen science projects. While initial citizen science applications in the Zooniverse focused on classifying and annotating anomalies across many astronomy images (e.g. Planet Hunters, http://www.planethunters.org ), the roster of applications continues to grow. Old Weather (http://www.oldweather.org ), for example, utilizes a simple transcription mechanism to collate temperature and other weather variables to determine past ocean climates. The project initially focused efforts on Royal Navy ship logs of the 20th century, but has since expanded to new sources of historic ship logs. The project, collaboratively developed by archivists, climate scientists, and citizen science experts has already transcribed over a million pages of such logs through engaging over 25,000 active volunteers since its start in 2010.

Notes from Nature is in many respects “experimental,” and is still in its prototype phase. Many different enhancements will be tested, such as badges. Rewarding users is a complex topic in citizen science, as many considerations need to be made about how it could affect the quality and accuracy of data being collected. In Notes from Nature, the primary role of badges is to bring attention to particular work or achievements that can be made by volunteers in topics or datasets of interest. Ultimately, this will build into a Zooniverse-wide badge system, allowing users can collect badges from multiple domains of citizen science work. Badges will be an ongoing development in Notes from Nature, and the tool itself is expected to go through further iteration and refinement long after its initial full public release in August 2012.

The current focus of Notes from Nature is on accurate transcription of data exactly as it is recorded in the non-digital version. The first release will offer no opportunities for interpretation or annotation. We will continue to improve the transcription tool built for each of the data sources and add new interfaces for users, including tools for improving the quality of data and fitness for use. Examples to be developed in the near future include performing taxonomic and geographic “referencing”. Taxonomic referencing would allow users to use services to check if names on labels are still valid, and if not, locate and provide an interpreted valid name (Thomer et al. 2012). Geographic referencing would provide means to convert textual locality descriptions into latitude, longitude, uncertainty triplets (Hill et al. 2009).

After Notes from Nature demonstrates that it works and is of wide interest, we hope grow our network of biocollections collaborators. We do so recognizing there is also a set of responsibilities to the community, including: 1) developing a reasonable and clear process for new biocollections to participate; 2) assuring that Notes From Nature does not overwhelm the community of citizen scientists with seemingly insurmountable tasks; 3) recognizing room for growth in this domain such that Notes From Nature can help address the needs of many citizen science transcription efforts. This challenge has been faced previously in Old Weather, where it is apparent that a much greater need for ledger transcription exists than was first thought. Our design architecture anticipates such growth, with Projects and Collections, built to facilitate local control of material coming from individual and partnering biocollections, and Missions, which target interests of citizen scientists and cut across any one project or collection.

Through Notes from Nature, we hope to team with citizen scientists to further widen the pipeline of digital biodiversity data for research. Both the application, and the new digitization it facilitates, may prove transformative for biological collections, citizen science and biodiversity science respectively. For biological collections and citizen scientists, we hope to bring new attention to those collections and the institutions that house them by connecting volunteers around the world to stories those data can tell. For biodiversity sciences, Notes from Nature will help unlock historical records that can help create and refine biodiversity baselines essential for documenting biodiversity change now and into the future.
